# Expression of aquaporins in bronchial tissue and lung parenchyma of patients with chronic obstructive pulmonary disease

**DOI:** 10.1186/2049-6958-9-29

**Published:** 2014-05-26

**Authors:** Carmen Calero, Jose Luis López-Campos, Lourdes Gómez Izquierdo, Rocío Sánchez-Silva, Jose Luis López-Villalobos, Francisco J Sáenz-Coronilla, Elena Arellano-Orden, Ana Montes-Worboys, Miriam Echevarría

**Affiliations:** 1 Unidad Médico-Quirúrgica de Enfermedades Respiratorias, Hospital Universitario Virgen del Rocio, Sevilla, Spain; 2 Instituto de Biomedicina de Sevilla (IBiS), Avda. Manuel Siurot, s/n 41013, Sevilla, Spain; 3 CIBER de Enfermedades Respiratorias (CIBERES), Instituto de Salud Carlos III, Madrid, Spain; 4 Departamento de Anatomía Patológica, Hospital Universitario Virgen del Rocio, Sevilla, Spain

**Keywords:** Aquaporin, Chronic obstructive pulmonary disease, Bronchial tissue, Lung parenchyma

## Abstract

**Background:**

Aquaporins AQP1 and AQP5 are highly expressed in the lung. Recent studies have shown that the expression of these proteins may be mechanistically involved in the airway inflammation and in the pathogenesis of chronic obstructive pulmonary disease (COPD). The aim of this study was to investigate the expression of AQP1 and AQP5 in the bronchial tissue and the lung parenchyma of patients with COPD and COPD-resistant smokers.

**Methods:**

Using a case–control design, we selected a group of 15 subjects with COPD and 15 resistant smokers (smokers without COPD) as a control, all of whom were undergoing lung resection surgery due to a lung neoplasm. We studied the expression of AQP1 and AQP5 in the bronchial tissue and the lung parenchyma by means of immunohistochemistry and reverse-transcription real-time polymerase chain reaction. Tissue expression of AQP1 and AQP5 was semi-quantitatively assessed in terms of intensity and expression by immunohistochemistry using a 4-point scale ranging from 0 (none) to 3 (maximum).

**Results:**

There were no significant differences in gene expression between COPD patients and resistant smokers both in the bronchial tissue and in the lung parenchyma. However, AQP1 gene expression was 2.41-fold higher in the parenchyma of smokers with COPD compared to controls, whereas the AQP5 gene showed the opposite pattern, with a 7.75-fold higher expression in the bronchus of smokers with COPD compared with controls. AQP1 and AQP5 proteins were preferentially expressed in endothelial cells, showing a higher intensity for AQP1 (66.7% of cases with an intensity of 3, and 93.3% of subjects with an extension of 3 among patients with COPD). Subtle interstitial disease was associated with type II pneumocyte hyperplasia and an increased expression of AQP1.

**Conclusions:**

This study provides pilot observations on the differences in AQP1 and AQP5 expression between COPD patients and COPD-resistant smokers. Our findings suggest a potential role for AQP1 in the pathogenesis of COPD.

## Background

Chronic obstructive pulmonary disease (COPD) is characterized by a chronic airflow limitation as a consequence of an enhanced inflammatory response to the inhalation of toxics and fumes, the most common one being tobacco smoke. Although tobacco smoke is the main risk factor for COPD, not all smokers develop the disease, and this group comprises the so-called resistant smokers. COPD is currently considered a major cause of morbidity and mortality worldwide [1] and has a devastating impact on health status. Much attention has been devoted to studying the expression of inflammatory markers in the lung to find biomarkers of diagnostic or prognostic value and to identify new potential therapeutic targets [2].

Aquaporins (AQPs) are a group of proteins discovered in the early 1990s that form specific channels for water transport across cells [3]. Several members of this family have been described in animals, yeasts, bacteria and plants, with at least 13 different AQPs in humans [4]. Among these proteins, AQP5 is most highly expressed in the lung. In addition to contributing to the water permeability of the cell membrane, AQPs participate in nerve signal transduction, skin flexibility, fat metabolism, membrane permeability to gases, and cell migration and proliferation [5]. Although no studies have assessed the expression of AQP5 in lung tissues from patients with COPD, a polymorphism in the AQP5 gene has been associated with lung function decline in COPD, the hallmark of disease progression. Taken together, these results suggest that the expression of this protein could be mechanistically involved in COPD pathogenesis or inflammatory expression [6].

The expression of AQP1 in lung carcinoma and pleural mesotheliomas has been recently studied. The findings indicated an increase in the expression of this protein in adenocarcinomas and mesotheliomas, but not in other lung carcinomas. Our results also showed high expression of AQP1 in the small blood vessels surrounding the tumor, suggesting an important role of AQP1 in tumor angiogenesis [7].

Despite these observations, the tissue expression of AQP1 and AQP5 in COPD patients is unknown. Using a case–control design, we selected a group of smokers with COPD and a group of COPD-resistant smokers, all of whom were undergoing lung resection surgery due to a lung neoplasm. The expression of AQP1 and AQP5 in the bronchial tissue and the lung parenchyma was investigated by means of immunohistochemistry and reverse-transcription real-time polymerase chain reaction (RT-qPCR). The tissue expression of AQP1 and AQP5 was then compared in patients with COPD and in COPD-resistant smokers.

## Methods

The present study was designed as an observational case–control pilot study. A total of 15 smokers with COPD and 15 COPD-resistant smokers undergoing lung surgery (lobectomy or pneumonectomy, due to a localized primary lung neoplasm, were enrolled. The study followed the tenets of the Declaration of Helsinki and was approved by our institutional review board. Written informed consent was obtained from all participants.

Patients > 40 years were consecutively selected until we obtained 15 cases per group on the day of admission before lung surgery. A detailed standardized questionnaire was administered to each patient to identify the clinical and functional diagnostic criteria for COPD. According to the international guidelines [8], patients with a tobacco history > 10 pack/years and a post-bronchodilator spirometry showing a ratio of forced expiratory volume in the first second (FEV_1_)/forced vital capacity (FVC) below 0.7 were considered COPD patients. By contrast, subjects with a FEV_1_/FVC ≥ 0.7 were considered resistant smokers. The severity of COPD was determined based on FEV_1_ expressed as the percentage of the predicted value, according to the Global Initiative for Obstructive Lung Disease (GOLD) guidelines [8]. Patients with any other lung disease associated with airflow obstruction or any chronic inflammatory disease were not enrolled in the study. Similarly, we excluded patients for whom the duration of the surgery (from the opening of the cutaneous layer until the extraction of the sample) exceeded three hours. Once the anatomical lobe was extracted, a portion of macroscopically healthy bronchus and parenchyma samples, distant from the primary lesions, was transferred to a sterile satellite table on ice and processed as described below. The bronchial tissue and the lung parenchyma were selected as the primary tissues involved in the pathogenesis of COPD.

### Gene expression

AQP gene expression was analyzed in the tissue samples by RT-qPCR, which included three phases: RNA extraction, reverse transcription to cDNA, and gene amplification by PCR. RNA was isolated from fresh tissue with a TriSure kit (Bioline, London, UK) according to the manufacturer’s protocol. The RNA was treated with RNase-free DNase I using a commercial kit (QIAgen GmbH, Hilden, Germany) to remove any residual genomic DNA that may have been present in the RNA. cDNA was synthesized using an iScript kit (Bio-Rad, Hercules, CA, USA). Each reaction was performed in duplicate using a final reaction volume of 25 μL: 2 μL of cDNA (40 ng/μl), 12.5 μL Sybr green PCR master mix (Stratagene, La Jolla, CA, USA) and 10.5 μL primers/H_2_O. RT-qPCR was performed using an MX3005P system (Stratagene) at 95°C for 30 sec, 60°C for 1 min, and 72°C for 30 sec. Gene amplification was normalized to 18S rRNA. Data analysis was performed using the 2^-∆∆Ct^ method [9]. The primers used are listed in Table 1.

**Table 1:** Primer sequences used in this study

	**Forward**	**Reverse**
18S rRNA	5′-TGAAATATCCAGAACATCTTA-3′	5′-GCAAAATTTATTGTCCCATCAT-3′
Aquaporin-1	5′-GGACACCTCCTGGCTATTGACTAC-3′	5′-GTTGCTGAAGTTGTGTGTGATCAC3′
Aquaporin-5	5′-CTGGCTGCCATCCTTTACTTCT-3′	5′-CCATGGTCTTCTTCCGCTCTT-3′

### Immunohistochemistry

Since all bronchial samples were utilized for gene expression studies, bronchial immunohistochemistry was not feasible in this study. Parenchymal samples examined were obtained from formalin-fixed, paraffin-embedded tissue. Tissue slices (5 μm) were cut with a microtome and mounted on microscope slides. The immunohistochemical procedure started with removal of the paraffin from tissue slices by immersion in xylene and rehydration through a series of decreasing dilutions of ethanol. Endogenous peroxidase activity was blocked by preincubation of slices in 3% H_2_O_2_. Heat-induced epitope retrieval for AQP1 was carried out by incubating tissue sections at 65°C for 1 h in 10 mM sodium citrate (pH 6). For AQP5, samples were heated under high pressure for 3 minutes in 4.5 mM Tris (pH 8) and 1 mM EDTA. Rabbit polyclonal anti-AQP1 (1:500 dilution; Abcam, Cambridge, UK) and rabbit polyclonal anti-AQP5 (1:50 dilution; Santa Cruz Biotechnology, Santa Cruz, CA, USA) antibodies were applied to the tissues. All specimens were then treated by the two-step EnVision plus Dual Link System-HRP (DakoCytomation, Dako, Glostrup, Denmark) procedure using goat anti-rabbit immunoglobulins conjugated to a peroxidase-labelled polymer and a DAB-substrate-DAB-chromogen to develop brown precipitates. Sections were photographed using an AX70-Olympus microscope equipped with an Olympus DP10 camera. No staining was observed when the primary antibody was omitted. Counter-staining was performed with haematoxylin, and the sections were analyzed by an experienced pathologist. Immunohistochemistry was semi-quantitatively evaluated by an experienced lung pathologist using a modified H-scoring system [10]. The intensity and the extent of the expression were evaluated in the tissue vessels, type II pneumocytes, and basal cells using a scale ranging from 0 (none) to 3 (maximum). The intensity was scored as follows: 0: no expression; 1: non-uniform and weak expression; 2: uniform but weak expression; 3: uniform and strong expression. The extent of expression was coded as 0: none; 1: focal expression (<33%); 2: zonal expression (33–66%); 3: diffuse expression (>66%).

### Statistical analysis

The statistical computations were carried out using the Statistical Package for the Social Sciences (SPSS, IBM Corporation, Somers, NY, USA) version 19.0. Categorical variables were described using the absolute and relative frequencies of their categories. Quantitative variables were expressed as means and standard deviations. The expression of the AQP1 and AQP5 genes both in the bronchial tissue and the lung parenchyma was compared between cases and controls using the Mann–Whitney U test. Comparisons between the two anatomical locations were performed with the Wilcoxon test. The differences in gene expression were graphically depicted using a bar plot representing the mean with standard error bars. The alpha error was set at 0.05 (two-tailed).

## Results

The sample included 15 COPD patients (93.3% males, mean age: 68 years, mean FEV_1_: 72%) and 15 resistant smokers (66.7% males, mean age 62 years, mean FEV_1_: 92%) as control. The general characteristics of the study participants are summarized in Table 2. Four COPD patients (26.7%) were in GOLD stage 1, whereas 11 (73.3%) were categorized as GOLD stage 2. In the COPD group, 4 (26.7%) patients were under treatment with a fixed-dose combination of an inhaled corticosteroid plus a long-acting β_2_ agonist, whereas 3 (20%) patients were receiving tiotropium. One patient was diagnosed with a neuroendocrine carcinoma, whereas another subject had an adenosquamous carcinoma. Two patients had a final pathological diagnosis of benign lung disease (lung adenoma, n = 1; and aspergilloma, n = 1).

**Table 2:** General characteristics of the study participants

	**Controls (n = 15)**	**COPD patients (n = 15)**	**p**
Male sex (n)	14 (93.3)	10 (66.7)	NS
Age (years)	62.5 (9.8)	68.3 (7.4)	NS
Active smokers (n)	3 (20)	3 (20)	NS
Body mass index (Kg/m^2^)	26.1 (4.4)	28.5 (4.2)	NS
FVC (mL)	3,274.1 (1,108.5)	3,016 (310.2)	NS
FVC (%)	92.8 (15.9)	86.7 (20.9)	NS
FEV_1_ (mL)	2,459.1 (775.5)	1,717 (574.6)	0.021
FEV_1_ (%)	92.8 (33.1)	72 (13)	0.031
FEV_1_/FVC (%)	76.2 (6.6)	62.5 (4.8)	< 0.001
Resection type			NS
- Pneumonectomy or (n)	4 (26.7)	2 (13.3)	
- Lobectomy (n)	11 (73.3)	12 (80.0)	
- Atypical (n)	0	1 (6.7)	
Pathology:			NS
- Squamous carcinoma (n)	6 (40.0)	8 (53.3)	
- Adenocarcinoma (n)	5 (33.3)	7 (46.7)	
- Other (n)	2 (13.3)	0	
- Benign tumor (n)	1 (6.7)	0	
- Non-neoplastic disease (n)	1 (6.7)	0	

Data are expressed as means (standard deviations) or absolute (relative) frequencies, as appropriate. FEV_1_, forced expired volume in 1 second; FVC, Forced vital capacity; NS, not significant.

### Gene expression

**Figure 1:**
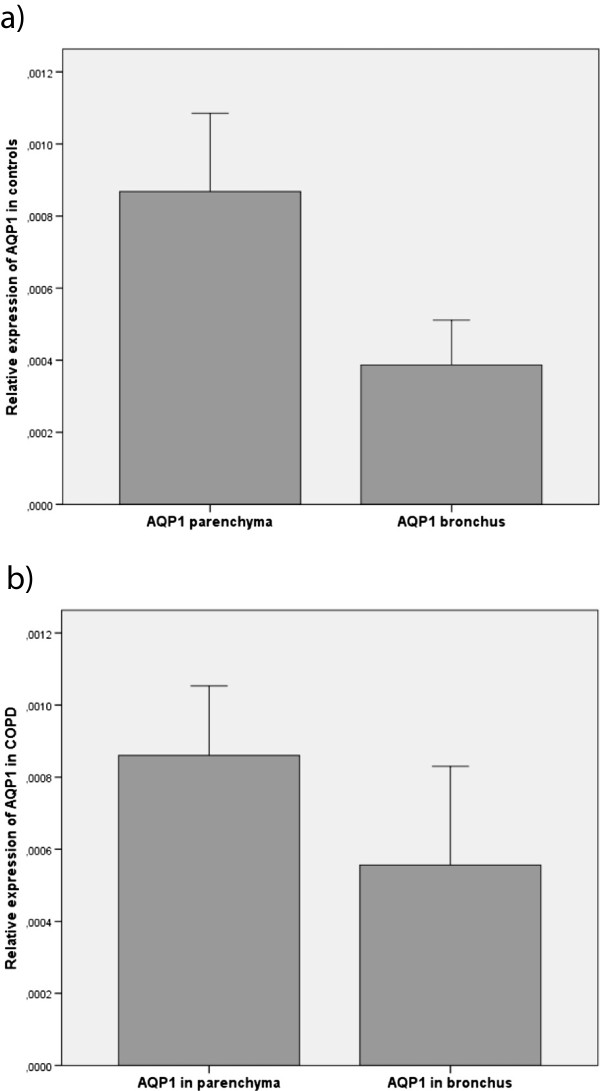
**Relative expression of AQP1 in the lung parenchyma as compared to the bronchus in COPD patients and controls. a)** Controls (p = 0.088); **b)** COPD (p = 0.035).

**Figure 2:**
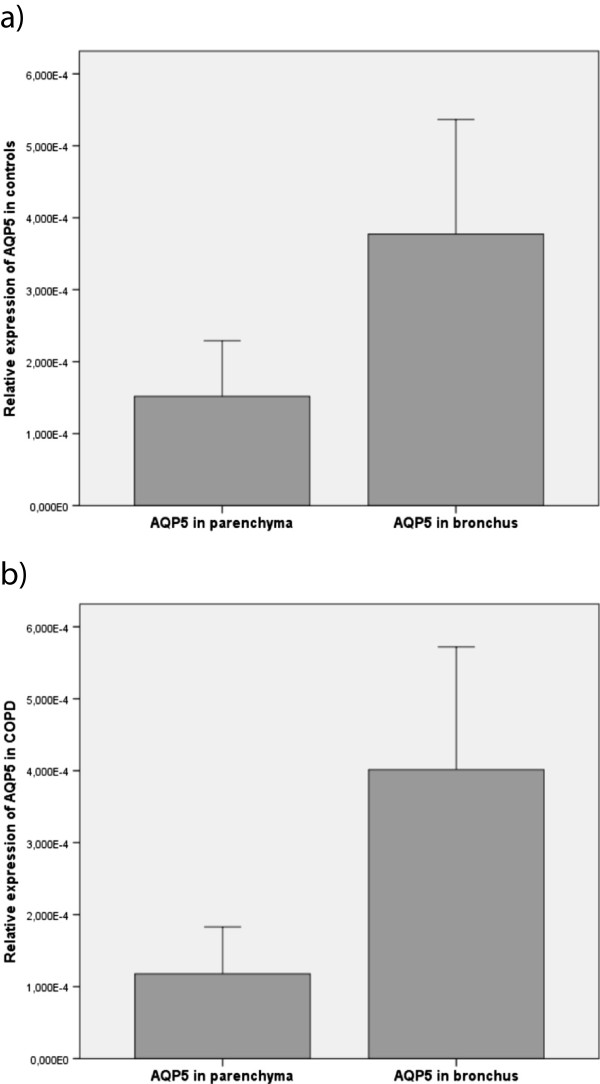
**Relative expression of AQP5 in the lung parenchyma as compared to the bronchus in COPD patients and controls. a)** Controls (p = 0.112); **b)** COPD (p = 0.023).

We found no differences in gene expression in either location between smokers with COPD and resistant smokers for both AQP1 and AQP5*.* When comparing the parenchymal expression with the bronchial one, we observed differences according to disease status. In the control group, the expression of AQP1 was 2.24-fold higher in the lung parenchyma than in the bronchus; a similar 2.22-fold increase was observed in COPD cases (Figure 1). In contrast, AQP5 gene expression showed an opposite pattern of expression, with higher levels in the bronchus (increases of 2.48-fold in controls and 19.4-fold in cases; Figure 2). We found no difference in gene expression between the cases receiving any treatment and those not receiving treatment or between active and non-active smokers. Similarly, gene expression was not significantly influenced by the GOLD severity stages of COPD or the underlying neoplasm.

### Immunohistochemistry

**Figure 3:**
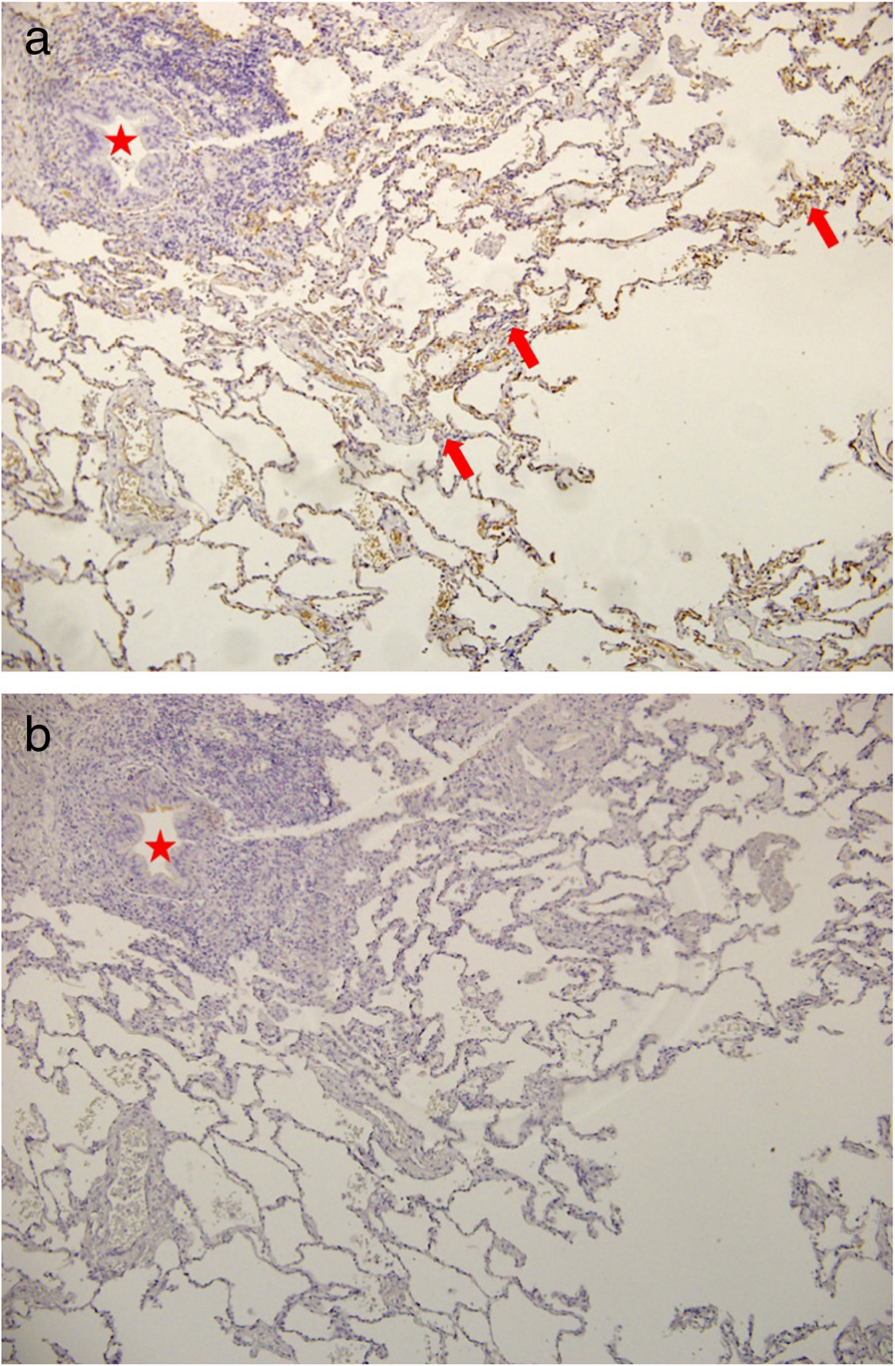
**Immunohistochemistry of the lung parenchyma for AQP1 (panel a) and AQP5 (panel b) in COPD patients.** The arrows indicate type 2 pneumocytes. The star shows a blood vessel.

AQP1 and AQP5 were preferentially expressed in the vessel wall, showing a higher intensity for AQP1. In cases where some subtle interstitial disease appeared, the areas of type 2 pneumocyte hyperplasia showed an increased AQP1 expression (Figure 3, Table 3). AQP5 was not intensely expressed in patients with COPD, except in basal cells, which showed a slight increase over the surrounding cells (Figure 3). There were no significant associations between the clinical parameters and the immunohistochemical expression of either AQP1 or AQP5.

**Table 3:** Immunohistochemistry for AQP1 and AQP5: number of subjects with maximal scores for extent and intensity

			**Controls (n = 15)**	**COPD (n = 15)**	p*
AQP1	Vessels	Intensity = 3	13 (86.7%)	10 (66.7%)	NS
		Extension = 3	14 (93.3%)	14 (93.3%)	NS
	Type 2 pneumocytes	Intensity = 3	4 (26.7%)	5 (33.3%)	NS
		Extension = 3	1 (6.7%)	8 (53.3%)	0.016
	Basal cells	Intensity = 3	0 (0%)	0 (0%)	NS
		Extension = 3	0 (0%)	0 (0%)	NS
AQP5	Vessels	Intensity = 3	7 (46.7%)	5 (33.3%)	0.094
		Extension = 3	3 (20%)	2 (13.3%)	NS
	Type 2 pneumocytes	Intensity = 3	1 (6.7%)	0 (0%)	NS
		Extension = 3	2 (13.3%)	2 (13.3%)	NS
	Basal cells	Intensity = 3	5 (33.3%)	3 (20%)	NS
		Extension = 3	5 (33.3%)	8 (53.3%)	NS

*χ^2^ test. Only p below 0.1 are shown in the Table. NS, not significant.

## Discussion

The present study describes the expression of both AQP1 and AQP5 in patients with COPD as compared to resistant smokers. We observed a significant overexpression of AQP1 in the parenchymal tissues from smokers with COPD. Both AQPs were mostly expressed in the vessel wall, with an increase in type 2 pneumocytes in smokers with COPD.

Cells expressing AQPs on their cell surface have up to 50-fold higher water permeability than cells that do not [11]. AQPs have important roles in the pathogenesis of pulmonary diseases, especially lung cancers. Recently, a mouse model of asthma induced by ovalbumin has been used to study the mRNA and protein expression of AQP1 and AQP5 [12]. The authors found that both AQPs were significantly increased by treatment with dexamethasone, ambroxol, and terbutaline, indicating that AQP1 and AQP5 are closely related to pulmonary edema but not to eosinophil infiltration or mucus secretion in patients with asthma. In the present study on COPD patients, we found an isolated increase in AQP1. Inflammation is qualitatively different between asthma and COPD, with a predominance of eosinophils in the former and neutrophils in the latter [13]. This different cell infiltration is not only a descriptive phenomenon, but it is also associated with a different therapeutic response, in particular to inhaled corticosteroids [14].

Some characteristics of the study participants deserve comment. In our sample of COPD patients, four subjects were taking inhaled corticosteroids. Although this makes our group not entirely homogeneous, the results of subgroup analyses did not reveal any significant differences in the expression of AQPs. Taken together, these findings indicate that inhaled corticosteroids did not have a major impact on AQP expression, at least in our sample. The lack of inclusion of non-smokers is an important caveat inherent in our study. As we included patients who underwent surgery because of a suspected malignancy, it is difficult to find never-smokers in this subject group. Finally, we cannot exclude the AQP expression may differ according to the specific COPD phenotype. In this regard, it has been recently shown that AQP5 may regulate cigarette smoke-induced emphysema by modulating the barrier and immune properties of the epithelium [15]. Future research on AQP expression in the airways should, therefore, include never-smokers and different COPD phenotypes.

There is only one interesting study from China showing that the expression of AQP5 is correlated with COPD. The authors found a correlation between a lower expression of AQP5 in the bronchial tissue (obtained by bronchoscopy) and an increase in the mucus secretion (as measured by MUC5AC gene expression) [16]. Notably, the results were confirmed by immunohistochemistry. These results highlight the importance of phenotyping for a correct interpretation of them. Unfortunately, an accurate patient phenotyping was not available in this study. In addition, we were unable to use a standardized questionnaire for the assessment of chronic sputum production because at the beginning of the study no specific *ad hoc* questionnaire was available [17].

In the present study, we did not find any significant differences in aquaporins expression according to the underlying neoplasm. Notably, we previously found AQP1 to be differentially expressed between lung adenocarcinomas and squamous cell carcinomas [7]. In this study, we sampled normal tissues localized as distant as possible from the primary lesion. Using such an approach, we were unable to find significant differences in both AQP1 and AQP5 expression according to the underlying neoplasm. These results suggest that the expression of aquaporins in the normal parenchyma is not directly influenced by the tumor type. This observation may be explained by the impact of specific genetic polymorphisms. Notably, previous studies have shown that variants in the AQP5 gene may influence the risk of developing COPD [18] and can have an impact on the rate of lung function decline in continuous smokers with COPD [6].

In the present study, we consistently observed an increased AQP1 expression in type 2 pneumocytes, especially in areas of subtle interstitial fibrosis. Despite being traditionally considered as two distinct conditions, growing evidence suggests that emphysema and fibrosis may closely intertwined [19,20]. Although some biomarkers (e.g., the KL-6 protein) have been associated with this phenotype, the potential role of AQP1 in this context remains to be determined [17]. Although the relation between fibrosis and emphysema was not the main focus of this study, we believe that the current findings may stimulate further studies on the potential role played by AQP1 expressed in type 2 pneumocytes in the pathogenesis of the fibrosis/emphysema phenotype.

Because most AQPs are constitutively expressed on the plasma membrane, the regulation of their function occurs mainly at the transcriptional level [4]. COPD is the result of a complex interplay of genetic and environmental factors [21]. Cigarette smoking is the major environmental determinant of COPD, and gene–smoking interactions have been associated with lung function in COPD and other chronic lung diseases [22]. We found that AQP1 expression was higher in the parenchyma of patients with COPD as compared to controls. In contrast, AQP5 was hyperexpressed in the bronchus of patients with COPD as compared to the controls. In this study, the medical treatment did not have a significant impact on AQP gene expression. Interestingly, AQP5 was not intensely expressed in the samples from patients with COPD, the only exception being the basal cells (which showed a slightly increased expression compared to the surrounding cells). It is noteworthy that a decreased expression of human AQP5 has been previously associated with an overproduction of mucus in the airways of subjects with COPD and a significant reduction in lung function [15].

## Conclusions

In conclusion, our observations provide preliminary evidence regarding the expression of AQP1 and AQP5 in patients with COPD as compared to COPD-resistant smokers. In particular, AQP1 may play a role in the pathogenesis of COPD. Further research is needed to confirm the clinical and biological relevance of these findings.

## Competing interests

The authors declare no competing interests. The authors did not receive reimbursements, fees, funding, or salary from any organization that may have a financial interest in the publication of this manuscript. Moreover, all authors do not hold any stocks or shares in an organization that may have a financial interest in relation to this manuscript. The authors do not have patent activities related to this manuscript.

## Authors’ contributions

All authors have contributed significantly to the study and have read and approved the submission of the manuscript in the current form. Moreover, they grant an exclusive license to the journal in the event of the work being accepted. EA and FJ SC carried out the molecular studies and performed sequence alignments; JLL-C and AMW participated in the design of the study, performed the statistical analysis, and drafted the manuscript; CCA conceived the study, participated in its design and coordination, recruited patients from the surgical waiting list, and drafted the paper; JLL-V collected the anatomical samples during surgery; LGI prepared biopsy specimens; RSS and ME performed immunohistochemistry.
